# A promising prognostic grading system incorporating weight loss and inflammation in patients with advanced cancer

**DOI:** 10.1002/jcsm.13376

**Published:** 2023-11-20

**Authors:** Xi Zhang, Jia‐Xin Huang, Meng Tang, Qi Zhang, Li Deng, Chun‐Hua Song, Wei Li, Han‐Ping Shi, Ming‐Hua Cong

**Affiliations:** ^1^ Department of Comprehensive Oncology National Cancer Center/National Clinical Research Center for Cancer/Cancer Hospital, Chinese Academy of Medical Sciences and Peking Union Medical College Beijing China; ^2^ Key Laboratory of Cancer FSMP for State Market Regulation Beijing China; ^3^ Beijing International Science and Technology Cooperation Base for Cancer Metabolism and Nutrition Beijing China; ^4^ Cancer Center of the First Hospital of Jilin University Changchun China; ^5^ Department of Gastrointestinal Surgery Zhejiang Cancer Hospital Hangzhou China; ^6^ Department of Gastrointestinal Surgery, Department of Clinical Nutrition, Beijing Shijitan Hospital Capital Medical University Beijing China; ^7^ Department of Epidemiology, College of Public Health Zhengzhou University Zhengzhou China

**Keywords:** Advanced cancer, Grading system, Inflammation, Prognosis, Weight loss

## Abstract

**Background:**

Involuntary weight loss and increased systemic response are frequently observed in patients with cancer, especially in advanced stages. This study aimed to develop a powerful weight loss and inflammation grading system (WLAIGS) and investigate its prognostic performance in patients with advanced cancer.

**Methods:**

This multicentre prospective cohort study included 11 423 patients with advanced cancer. A 4 × 4 matrix representing four different per cent weight loss (WL%) categories within each of the four different neutrophil‐to‐lymphocyte ratio (NLR) categories (16 possible combinations of WL% and NLR) was constructed. The WLAIGS consisted of four grades, with hazard ratios (HRs) for overall survival (OS) gradually increasing from grade 1 to grade 4. Survival analyses, including Kaplan–Meier curve, Cox proportional hazards regression, and sensitivity analysis, were performed to investigate the association between WLAIGS and OS. The secondary outcomes were short‐term survival, malnutrition, and quality of life. Two internal validation cohorts with a 7:3 ratio were used to validate the results.

**Results:**

The median age of patients with advanced cancer in our study was 59.00 (interquartile range, 50.00–66.00) years. There were 6877 (60.2%) and 4546 (39.8%) male and female participants, respectively. We totally recorded 5046 death cases during the median follow‐up of 17.33 months. The Kaplan–Meier curve showed that the survival rate decreased from grade 1 to grade 4 in patients with advanced cancer (log‐rank *P* < 0.001). The WLAIGS was an independent risk factor associated with OS adjusting for confounders, with HRs increasing from 1.20 (95% confidence interval (CI), 1.11–1.29; *P* < 0.001) in grade 2, 1.48 (95% CI, 1.38–1.60; *P* < 0.001) in grade 3 to 1.73 (95% CI, 1.58–1.89; *P* < 0.001) in grade 4. In each weight loss% group (2.5 ≤ WL% < 6.0; 6.0 ≤ WL% < 11.0, WL% ≥ 11.0), a NLR above 3 was associated with shorter survival and served as an independent prognostic predictor. The risk of short‐term mortality, malnutrition, and poor quality of life increased with WLAIGS grade. Two internal validation cohorts confirmed that the WLAIGS independently identified the survival of patients with advanced cancer.

**Conclusions:**

The WLAIGS, which reflects malnutrition and systemic inflammation status, is a robust and convenient tool for predicting the prognosis of patients with advanced cancer.

## Introduction

Weight loss is commonly observed in patients with advanced or incurable cancer, with frequencies ranging from 31% to 87% for different tumour types.^1^, ^2^ It appears more often in patients with solid tumours, especially gastrointestinal, pancreatic, and lung cancers.^3^ Involuntary weight loss is associated with reduced anti‐cancer treatment efficacy,^4^ poorer quality of life (QoL), and shortened survival interval^2^ and is considered a good prognostic factor in various cancers.^5^, ^6^ The prognostic impact of weight loss on survival is independent of disease, site, stage, or performance score.^7^ Moreover, it is a hallmark of cancer‐related malnutrition and a pivotal parameter in the diagnosis of cancer cachexia.^2^ Various factors, including decreased food intake, metabolic alterations, and adverse effects of anti‐cancer treatments, contribute to cancer‐related weight loss.^8^, ^9^ Metabolic alterations are characterized by tumour‐derived catabolic factors and inflammation, which promote skeletal muscle and adipose tissue catabolism, increase energy expenditure, and inhibit nutrient utilization.^10^ In addition, the active systemic inflammatory response causes anorexia and catabolism, which ultimately leads to loss of body weight, alterations in body composition, and a decline in physical function.^11^

Since Coussens et al. first discovered leukocytes within tumour cells and hypothesized that inflammation increases cellular proliferation, it has been widely accepted as a hallmark feature of the development and progression of cancer.^12^, ^13^ There is a great deal of evidence on the association between an elevated systemic inflammatory response and poor survival. The neutrophil‐to‐lymphocyte ratio (NLR) is a convenient laboratory parameter that reflects inflammatory status in clinical practice because of its accessibility from routine blood tests. An increased NLR is correlated with a higher tumour stage.^14^ In 2005, Walsh et al. first investigated the predictive value of NLR in colorectal cancer and confirmed NLR as an effective prognostic predictor.^15^ In recent years, NLR has also been reported as a powerful prognostic factor in various solid tumours, including non‐small cell lung,^16^ breast,^17^ liver,^18^ and gastric^19^ cancers.

Because systemic inflammation is a major contributor to involuntary weight loss, patients with the same level of body weight loss may have different systemic inflammation statuses, which may lead to different prognostic outcomes. Therefore, the present study aimed to develop a novel and convenient prognostic grading system that incorporates weight loss and inflammatory burden in patients with advanced cancer. We comprehensively investigated its value in predicting survival in patients with advanced carcinoma as well as its relationship with short‐term survival, malnutrition, and QoL.

## Methods

### Study participants and design

This multicentre prospective cohort study included patients from the Investigation on Nutrition Status and its Clinical Outcomes of Common Cancers project in China (registration number: ChiCTR1800020329).^20^ All patients were admitted and received anti‐cancer treatments, including surgery, chemotherapy, and radiotherapy. The inclusion criteria were as follows: (a) age >18 years, (b) pathological diagnosis of a solid tumour, (c) advanced tumour stage (stage III and IV), and (d) complete record of weight within 6 months. The exclusion criteria were as follows: (a) presence of active infection or severe systemic immunodeficiency disease, (b) lack of important data required for analysis, and (c) weight gain (owing to several forms of weight gain, such as oedema, ascites, increased organ volume, and tumour mass). A flowchart of the study is shown in Figure S1. This study was conducted in accordance with the principles outlined in the Declaration of Helsinki and approved by the ethics committees of all participating institutions. Written informed consent was obtained from all participants.

### Data collection

During the patient's hospital stay, expertized personnel were employed to collect baseline characteristics, including age, sex, history of weight loss, comorbidities (diabetes and hypertension), alcohol consumption, smoking status, type of cancer, tumour/node/metastasis (TNM) stage, treatment (e.g., surgery, chemotherapy, and radiotherapy), Nutrition Risk Screen 2002 (NRS 2002), Eastern Cooperative Oncology Group (ECOG) performance status, Patient‐Generated Subjective Global Assessment (PG‐SGA), body mass index (BMI), hand grip strength (HGS), calf circumference (CC), white blood cell, neutrophil, lymphocyte, and NLR. Pathological staging was performed according to the eighth edition of the American Joint Committee on Cancer TNM staging system. Patients with the score of NRS 2002 ≥ 3 were in the high nutritional risk group.^21^ The Eastern Cooperative Oncology Group performance status (ECOG‐PS) was converted using the Karnofsky performance status (KPS) as follows: ECOG‐PS 0 (KPS, 100), ECOG‐PS 1 (KPS, 90–80), ECOG‐PS 2 (KPS, 70–60), ECOG‐PS 3 (KPS, 50–40), and ECOG‐PS 4 (KPS, 30–0).^22^ The BMI was calculated as weight in kilograms divided by height in meters squared (kg/m^2^). HGS was measured in the nondominant hand using an electronic handgrip dynamometer. The patients were placed in the supine position with 90° knee flexion to measure the CC.

### Construction of weight loss and inflammation grading system (WLAIGS)

The per cent weight loss was calculated as follows: ([current weight in kilograms‐previous weight in kilograms]/previous weight) × 100. Previous weight was defined as the weight 6 months ago. If a 6 months weight was missing, the weight measured at 1, 2, or 3 months ago was used. The per cent weight loss was divided into four groups with cut‐off values of 2.5, 6.0, and 11.0 in accordance to study from Martin et al.^23^ Each group was then divided into four subgroups based on NLR values (cut‐off values: 3, 5, and 10).^24^ A 4 × 4 matrix representing four different per cent weight loss categories within each of the four NLR categories (16 possible combinations of WL and NLR) was constructed. Hazard ratios (HRs) for survival were calculated with reference to the group with weight loss of <2.5%, NLR of ≤3, and clustered approximate survival risk. The WLAIGS consists of four grades (1–4), with grade 1 having the lowest risk (longer survival) and grade 4 having the highest HR (shorter survival).

### Outcomes of present study

Telephone follow‐up surveys, periodic re‐examinations, and readmissions were conducted to collect the clinical outcomes for all patients. The primary outcome of our study was overall survival (OS), defined as the interval between the first assessment in the clinic and the date of death, date of withdrawal from the study, end of follow‐up (October 30, 2020), or time of last contact, whichever came first. The secondary outcomes were short‐term outcomes, malnutrition, and QoL. Short‐term outcome refers to the prognostic outcome of the patient within 3 months of treatment. Patients with a score of PG‐SGA ≥ 4 were considered as malnourished.^22^ QoL was evaluated using the 30‐item European Organization for Research and Treatment of Cancer Quality of Life Questionnaire, version 3.0 (QLQ‐C30). This questionnaire consists of five multi‐item functional scales (physical, role, social, emotional, and cognitive functions), three multiitem symptom scales (fatigue, pain and nausea & vomiting), six single‐item symptom scales (dyspnoea, insomnia, appetite loss, constipation, diarrhoea, and financial impact), and a two‐item Global QoL scale.^25^ The summary score of QLQ‐C30 was calculated as follows: summary score = ([physical functioning + role functioning + social functioning + emotional functioning + cognitive functioning] + [100‐fatigue] + [100‐pain] + [100‐nausea & vomiting] + [100‐dyspnoea] + [100‐insomnia] + [100‐appetite loss] + [100‐constipation] + [100‐diarrhoea])/13.^26^

### Statistical analysis

Statistical analyses were performed using the R software version 4.0.3. Continuous variables were expressed as medians (interquartile range [IQR]), and categorical variables were expressed as absolute numbers or percentages. The Cox proportional hazards model was used to assess the prognostic risk of the 16 groups cross‐classified according to per cent weight loss and NLR. Groups were classified into four grades based on the approximate HRs. The Kaplan–Meier method generated by the log‐rank test was used to assess the prognostic discrimination performance of WLAIGS. Univariate and multivariate Cox regression analyses were performed to identify the independent predictors of poor OS using HRs and 95% confidence intervals (CIs). Sensitivity analysis was conducted using repeated survival analyses, excluding patients who died within 6 months of the beginning of the study. Logistic regression analysis was used to assess the association between the WLAIGS and short‐term survival, malnutrition, and QoL. Two internal validation cohorts at a ratio of 7:3 were used to validate the prognostic value of WLAIGS. Statistical significance was defined as a two‐tailed *P* value of <0.05.

## Results

### Baseline characteristics of patients

**FIGURE 1:**
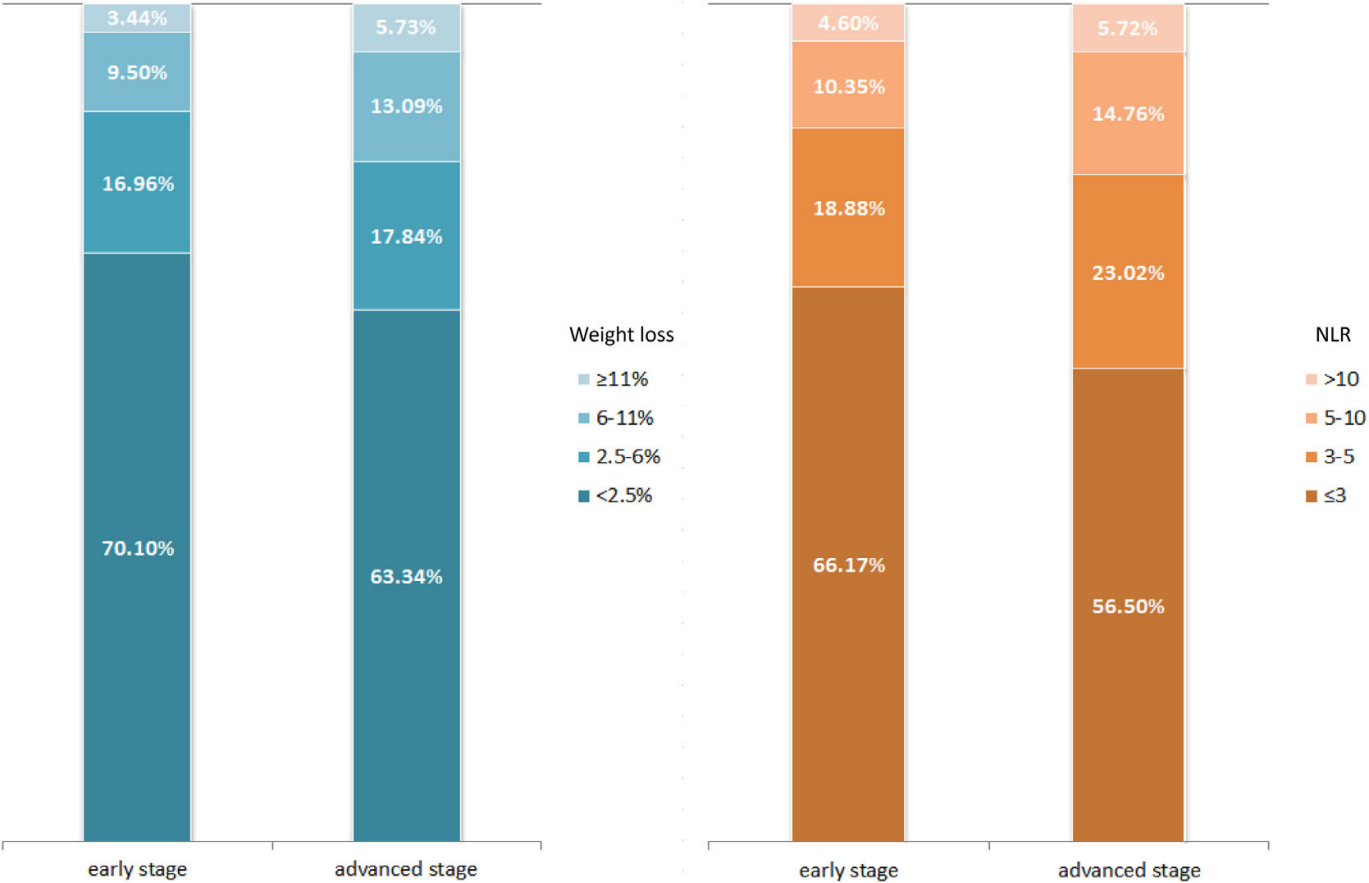
Distribution of weight loss% and NLR in early and advanced cancer patients. NLR, neutrophils to lymphocyte ratio.

A total of 11 423 patients diagnosed with advanced cancer were eligible for our study, with 4570 (40.0%) patients in stage III and 6853 (60.0%) patients in stage IV. The median age of all patients was 59.00 years (IQR, 50.00–66.00). There were 6877 (60.2%) male and 4546 (39.8%) female participants, respectively. The tumours were most frequently located in the lungs (25.8%), followed by the upper gastrointestinal tract (19.6%), colorectal (18.9%), and hepatobiliary and pancreatic (5.0%). The number of patients with WLAIGS grades 1 to 4 was 4951, 2854, 2270, and 1348, respectively. Detailed baseline characteristics are shown in Table 1. Compared with patients with early‐stage cancer, we found that patients with advanced cancer had a higher percentage of weight loss and higher NLR (Figure 1). The proportion of advanced patients experiencing ≥6% weight loss was 18.82%, which was higher than that of early patients (12.94%). There were 43.50% of advanced patients with NLR values above 3, whereas the percentage of early patients was only 33.83%.

**TABLE 1:** Patients' baseline characteristics stratified by WLAIGS

Variables	All patients (*n* = 11 423)	Grade 1 (*n* = 4951)	Grade 2 (*n* = 2854)	Grade 3 (*n* = 2270)	Grade 4 (*n* = 1348)	*P* value
Age, years	59.00 (50.00–66.00)	58.00 (49.00–65.00)	60.00 (51.00–66.00)	60.00 (51.00–67.00)	60.00 (51.00–68.00)	<0.001*
Gender (male/female)	6877/4546 (60.2%/39.8%)	2793/2158 (56.4%/43.6%)	1773/1081 (62.1%/37.9%)	1432/838 (63.1%/36.9%)	879/469 (65.2%/34.8%)	<0.001*
Smoking (yes/no)	5137/6286 (45.0%/55.0%)	2083/2868 (42.1%/57.9%)	1318/1536 (46.2%/53.8%)	1056/1214 (46.5%/53.5%)	680/668 (50.4%/49.6%)	<0.001*
Drinking (yes/no)	2346/9077 (20.5%79.5%)	920/4031 (18.6%/81.4%)	623/2231 (21.8%/78.2%)	459/1811 (20.2%/79.8%)	344/1004 (25.5%/74.5%)	<0.001*
Diabetes (yes/no)	975/10448 (8.5%/91.5%)	383/4568 (7.7%/92.3%)	260/2594 (9.1%/90.9%)	216/2054 (9.5%/90.5%)	116/1232 (8.6%/91.4%)	0.045*
Hypertension (yes/no)	2010/9413 (17.6%/82.4%)	771/4180 (15.6%/84.4%)	551/2303 (19.3%/80.7%)	439/1831 (19.3%/80.7%)	249/1099 (18.5%/81.5%)	<0.001*
Type of cancer						
Lung	2951 (25.8%)	1174 (23.7%)	691 (24.2%)	722 (31.8%)	364 (27.0%)	
Upper gastrointestinal	2236 (19.6%)	780 (15.8%)	671 (23.5%)	422 (18.6%)	363 (26.9%)	
Hepatobiliary and pancreatic	571 (5.0%)	153 (3.1%)	158 (5.5%)	148 (6.5%)	112 (8.3%)	
Colorectal	2159 (18.9%)	865 (17.5%)	602 (21.1%)	437 (19.3%)	255 (18.9%)	
Others	3506 (30.7%)	1979 (40.0%)	732 (25.6%)	541 (23.8%)	254 (18.8%)	
TNM stage						<0.001*
III	4570 (40.0%)	2247 (45.4%)	1150 (40.3%)	816 (35.9%)	357 (26.5%)	
IV	6853 (60.0%)	2704 (54.6%)	1704 (59.7%)	1454 (64.1%)	991 (73.5%)	
Surgery (yes/no)	4342/7081 (38.0%/62.0%)	1829/3122 (36.9%/63.1%)	1195/1659 (41.9%/58.1%)	821/1449 (36.2%/63.8%)	497/851 (36.9%/63.1%)	<0.001*
Radiotherapy (yes/no)	1685/9738 (14.8%/85.2%)	573/4378 (11.6%/88.4%)	367/2487 (12.9%/87.1%)	426/1844 (18.8%/81.2%)	319/1029 (23.7%/76.3%)	0.007*
Chemotherapy (yes/no)	5632/5791 (49.3%/50.7%)	2472/2479 (49.9%/50.1%)	1458/1396 (51.1%/48.9%)	1077/1193 (47.4%/52.6%)	625/723 (46.4%/53.6%)	<0.001*
NRS2002 (<3/≥3)	7394/4029 (64.7%/35.3%)	4038/913 (81.6%/18.4%)	1613/1241 (56.5%/43.5%)	1390/880 (61.2%/38.8%)	353/995 (26.2%/73.8%)	<0.001*
ECOG grade (≤1/>1)	5058/6365 (44.3%/55.7%)	2644/2307 (53.4%/46.6%)	1274/1580 (44.6%/55.4%)	836/1434 (36.8%/63.2%)	304/1044 (22.6%/77.4%)	<0.001*
PGSGA (<4/≥4)	3698/7725 (32.4%/67.6%)	2391/2560 (48.3%/51.7%)	783/2071 (27.4%/72.6%)	472/1798 (20.8%/79.2%)	52/1296 (3.9%/96.1%)	<0.001*
Body mass index, kg/m^2^	22.04 (19.83–24.30)	22.76 (20.58–24.92)	21.69 (19.58–24.03)	22.04 (20.00–24.17)	19.97 (17.97–22.22)	<0.001*
Calf circumference, cm	32.80 (30.35–35.00)	33.20 (31.00–36.00)	32.50 (30.00–35.00)	32.50 (30.00–35.00)	31.00 (28.58–33.00)	<0.001*
Hand grip strength, kg	24.00 (17.80–31.00)	25.00 (18.90–32.30)	23.80 (17.80–30.60)	23.60 (17.30–30.30)	21.10 (14.70–28.00)	<0.001*
White blood cell, ×10^9^/L	6.10 (4.70–7.94)	5.50 (4.34–6.80)	5.80 (4.50–7.43)	7.56 (5.77–9.84)	8.14 (6.07–11.25)	<0.001*
Neutrophil, ×10^9^/L	3.85 (2.71–5.50)	3.12 (2.34–4.02)	3.68 (2.60–5.10)	5.69 (4.20–7.50)	6.49 (4.60–9.16)	<0.001*
Lymphocyte, ×10^9^/L	1.40 (1.00–1.84)	1.69 (1.30–2.10)	1.40 (1.09–1.80)	1.04 (0.76–1.38)	0.90 (0.60–1.20)	<0.001*
NLR	2.69 (1.80–4.43)	1.91 (1.46–2.41)	2.89 (1.88–3.69)	5.35 (3.98–7.25)	7.17 (5.16–11.12)	<0.001*

The data were analysed using the Kruskal–Wallis test to determine the differences between the groups.

ECOG, Eastern Cooperative Oncology Group; NLR, neutrophils to lymphocyte ratio; NRS 2002, Nutrition Risk Screen 2002; PG‐SGA, Patient‐Generated Subjective Global Assessment; TNM, tumour/node/metastasis; WLAIGS, weight loss and inflammation grading system.

### Survival analysis of the weight loss and inflammation grading system

**FIGURE 2:**
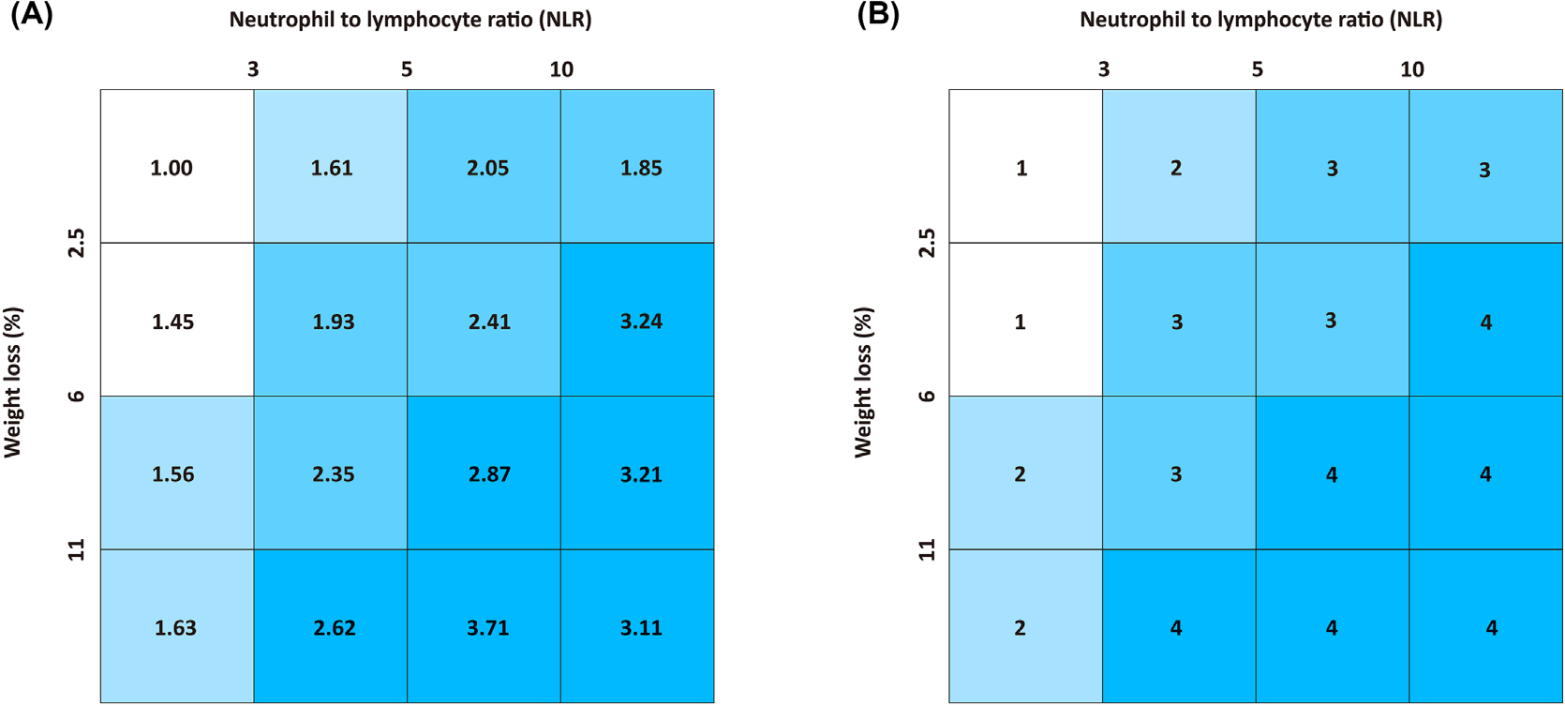
A 4 × 4 matrix incorporating four different weight loss% and NLR.

**FIGURE 3:**
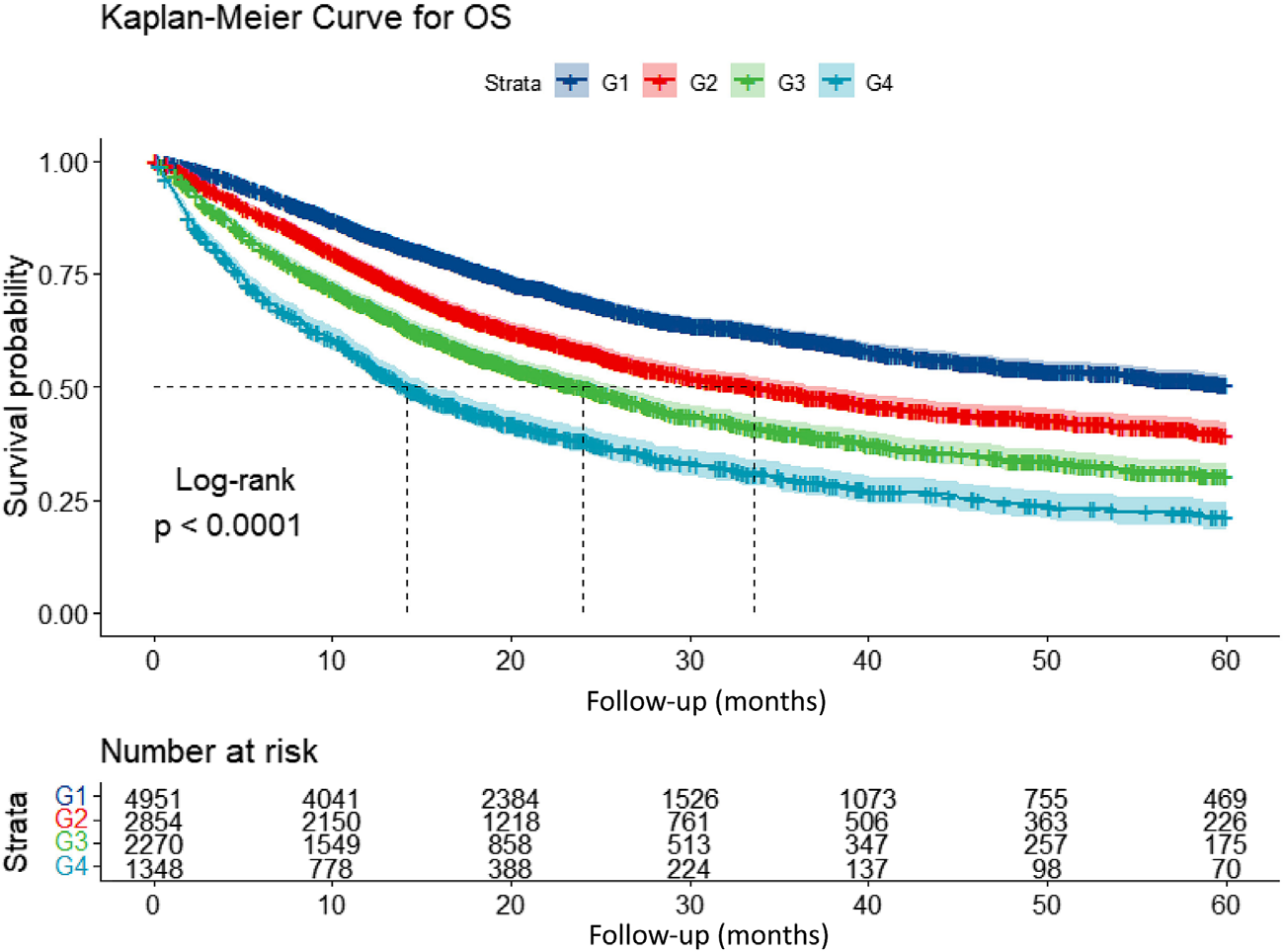
Kaplan–Meier curve of WLAIGS in advanced cancer patients. Abbreviation: WLAIGS: Weight loss and inflammation grading system; OS, overall survival.

**FIGURE 4:**
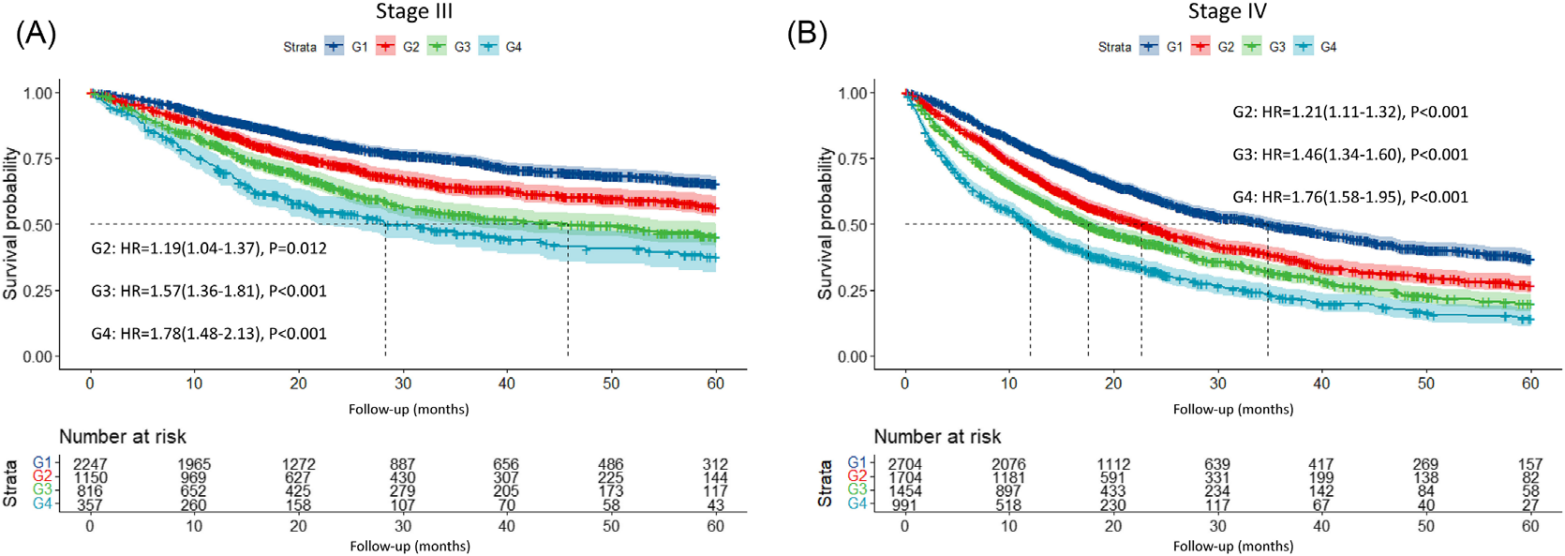
The prognostic value of WLAIGS for cancer patients in stage III and IV. Abbreviation: HR: Hazard ratio. Adjust for variables found significant at *P* < 0.05 in the univariate analyses.

A total of 5046 death cases occurred during a median follow‐up of 17.33 months. We calculated the HRs for OS of the 16 groups with reference to Group 1 (weight loss% < 2.5 and NLR ≤ 3) and clustered the groups with approximate HRs into one grade (Figure 2). Finally, we developed a four grades cancer related prognostic grading system incorporating per cent weight loss and NLR (Table S1). The Kaplan–Meier curve showed a significant step‐down survival probability deteriorating from grade 1 to grade 4 (log‐rank *P* < 0.001) (Figure 3). High‐grade WLAIGS was an independent risk factor for survival after adjusting for age, sex, smoking, drinking, diabetes, hypertension, tumour type, TNM stage, anti‐cancer treatments, NRS 2002, ECOG, BMI, HGS, and CC (Table 2). Compared with grade 1, the HRs reached to 1.20 (95% CI, 1.11–1.29; *P* < 0.001), 1.48 (95% CI, 1.38–1.60; *P* < 0.001), and 1.73 (95% CI, 1.58–1.89; *P* < 0.001) in grade 2, 3, and 4, respectively. In addition, we performed subgroup analysis stratified by TNM stage and found the WLAIGS had a significant prognostic performance for patients in both stage III (G2: HR, 1.19; 95% CI, 1.04–1.37; *P* = 0.012; G3: HR, 1.57; 95% CI, 1.36–1.81; *P* < 0.001; G4: HR, 1.78; 95% CI, 1.48–2.13; *P* < 0.001) and IV (G2: HR, 1.21; 95% CI, 1.11–1.32; *P* < 0.001; G3: HR, 1.46; 95% CI, 1.34–1.60; *P* < 0.001; G4: HR, 1.76; 95% CI, 1.58–1.95; *P* < 0.001) (Figure 4). In tumour‐specific analysis, a similar survival trend from grades 1 to 4 was observed in lung, upper gastrointestinal, hepatobiliary, pancreatic, and colorectal cancers. The prognostic predictive value of WLAIGS was better in advanced patients with lung cancer (G2: HR, 1.18; 95% CI, 1.04–1.34; *P* = 0.010; G3: HR, 1.46; 95% CI, 1.29–1.65; *P* < 0.001; G4: HR, 1.65; 95% CI, 1.40–1.94; *P* < 0.001) (Figure S2). Sensitivity analysis, which excluded 1491 patients who died within 6 months, validated the prognostic performance of WLAIGS in patients with advanced cancer (Figure S3).

**TABLE 2:** Univariate and multivariate Cox regression analyses associated with OS

Variables	No. of patients	Univariate		Multivariate	
		Hazard ratio (95% CI)	*P* value	Hazard ratio (95% CI)	*P* value
Age, years					
≤65	8320	Reference			
>65	3103	1.52 (1.44–1.62)	<0.001*	1.23 (1.16–1.31)	<0.001*
Gender					
Female	4546	Reference			
Male	6877	1.40 (1.32–1.48)	<0.001*	1.11 (1.03–1.20)	0.007*
Smoking					
No	6286	Reference			
Yes	5137	1.41 (1.33–1.49)	<0.001*	1.09 (1.02–1.18)	0.015*
Drinking					
No	9077	Reference			
Yes	2346	1.27 (1.19–1.35)	<0.001*	1.07 (0.99–1.15)	0.081
Diabetes					
No	10 448	Reference			
Yes	975	1.22 (1.11–1.34)	<0.001*	1.10 (1.00–1.21)	0.050
Hypertension					
No	9413	Reference			
Yes	2010	1.10 (1.03–1.18)	0.003*	0.95 (0.88–1.02)	0.164
Type of cancer					
Lung	2951	Reference			
Upper gastrointestinal	2236	0.88 (0.82–0.94)	<0.001*	0.98 (0.91–1.07)	0.703
Hepatobiliary and pancreatic	571	1.19 (1.06–1.34)	<0.001*	1.23 (1.09–1.39)	<0.001*
Colorectal	2159	0.50 (0.46–0.54)	<0.001*	0.63 (0.58–0.69)	<0.001*
Others	3506	0.32 (0.29–0.34)	<0.001*	0.42 (0.39–0.46)	<0.001*
TNM stage					
III	4570	Reference			
IV	6853	2.34 (2.20–2.49)	<0.001*	1.94 (1.82–2.07)	<0.001*
Surgery					
No	7081	Reference			
Yes	4342	0.82 (0.77–0.87)	<0.001*	0.88 (0.82–0.94)	<0.001*
Radiotherapy					
No	9738	Reference			
Yes	1685	1.10 (1.02–1.19)	0.010*	1.06 (0.98–1.15)	0.128
Chemotherapy					
No	5791	Reference			
Yes	5632	1.25 (1.19–1.33)	<0.001*	1.30 (1.23–1.38)	<0.001*
NRS2002					
<3	7394	Reference			
≥3	4029	1.65 (1.56–1.74)	<0.001*	1.12 (1.05–1.20)	0.001*
ECOG grade					
≤1	5058	Reference			
>1	6365	1.86 (1.75–1.97)	<0.001*	1.47 (1.38–1.56)	<0.001*
Body mass index, kg/m^2^					
High (≥18.5)	9843	Reference			
Low (<18.5)	1580	1.50 (1.39–1.61)	<0.001*	1.01 (0.92–1.11)	0.806
Hand grip strength, kg					
High (≥13.9)	9968	Reference			
Low (<13.9)	1455	1.51 (1.40–1.62)	<0.001*	1.32 (1.22–1.43)	<0.001*
Calf circumference, cm					
High (≥29.9)	9294	Reference			
Low (<29.9)	2129	1.47 (1.38–1.57)	<0.001*	1.09 (1.01–1.17)	0.029*
Score					
G1	4951	Reference			
G2	2854	1.45 (1.35–1.56)	<0.001*	1.20 (1.11–1.29)	<0.001*
G3	2270	1.93 (1.79–2.08)	<0.001*	1.48 (1.38–1.60)	<0.001*
G4	1348	2.78 (2.56–3.02)	<0.001*	1.73 (1.58–1.89)	<0.001*

The Wald test was used to test the significance of each variable included in the Cox proportional hazards model. Variables significant at *P* < 0.05 in the univariate analyses entered into multivariate Cox regression analysis, including age, gender, smoking, drinking, diabetes, hypertension, type of cancer, TNM stage, chemotherapy, radiotherapy, surgery, NRS 2002, ECOG, BMI, HGS, and CC.

ECOG, Eastern Cooperative Oncology Group; NRS 2002, Nutrition Risk Screen 2002; PG‐SGA, Patient‐Generated Subjective Global Assessment; TNM, tumour/node/metastasis; WLAIGS, weight loss and inflammation grading system.

### Comparison of survival between weight loss with and without inflammation

Kaplan–Meier curves showed that patients without inflammation had significantly longer survival than those with inflammation in the same per cent weight loss group (Figure S4). An inflammatory status with an NLR > 3 was an independent unfavourable factor associated with survival in patients with advanced cancer after adjusting for confounders (Table 3). In the group with the per cent of weight loss ranging from 2.5 to 6.0, the HR was 1.24 (95% CI, 1.08–1.43; *P* = 0.003) in patients with inflammation. The survival risk of patients with inflammation was 1.35 (95% CI, 1.18–1.55; *P* < 0.001) times higher than that of patients without inflammation in the group with per cent weight loss ranging from 6.0 to 11.0. In patients with per cent weight loss equal to or above 11.0, the HR for patients with inflammation rose to 1.49 (95% CI, 1.27–1.75; *P* < 0.001) compared with those without inflammation.

**TABLE 3:** Survival comparisons between patients with/without inflammation in same weight loss group

Weight loss%		Crude model		Adjusted model^a^		Adjusted model^b^	
		HR (95% CI)	*P* value	HR (95% CI)	*P* value	HR (95% CI)	*P* value
[2.5, 6]	NLR ≤ 3	Reference		Reference		Reference	
	NLR>3	1.63 (1.42–1.87)	<0.001*	1.44 (1.26–1.66)	<0.001*	1.24 (1.08–1.43)	0.003*
[6, 11]	NLR ≤ 3	Reference		Reference		Reference	
	NLR>3	1.64 (1.43–1.87)	<0.001*	1.42 (1.25–1.62)	<0.001*	1.35 (1.18–1.55)	<0.001*
≥11	NLR ≤ 3	Reference		Reference		Reference	
	NLR>3	1.78 (1.53–2.07)	<0.001*	1.63 (1.40–1.90)	<0.001*	1.49 (1.27–1.75)	<0.001*

The Wald test was used to test the significance of each variable included in the Cox proportional hazards model.

CI, confidence interval; HR, hazard ratio.

^a^ Adjusted for age, sex, TNM stage and tumour type.

^b^ Adjusted for age, sex, smoking, drinking, diabetes, hypertension, tumour type, TNM stage, treatments, NRS 2002, ECOG, BMI, CC, and HGS.

### Association between WLAIGS with secondary outcomes

In our study, 769 patients with advanced cancer died within 90 days of enrolment. In the logistic analysis, we found that the WLAIGS was an independent risk factor for short‐term survival after adjusting for confounders (Table 4). The risk of dying within 90 days rose to more than 1 (odds ratio [OR], 1.74; 95% CI, 1.37–2.22; *P* < 0.001), 2 (OR, 2.79; 95% CI, 2.22–3.52; *P* < 0.001) and 3 (OR, 3.87; 95% CI, 3.02–4.98; *P* < 0.001) times in grade 2 to 4 with reference to grade 1 (P for trend <0.001). A total of 7725 participants were diagnosed with malnutrition. A high‐grade WLAIGS was independently associated with the prevalence of malnutrition. The ORs of developing malnutrition in grades 2, 3 and 4 were 1.49 (95% CI, 1.33–1.68; *P* < 0.001), 2.34 (95% CI, 2.06–2.67; *P* < 0.001) and 7.16 (95% CI, 5.35–9.79; *P* < 0.001), respectively, with *P* < 0.001 for trend. The QoL was evaluated using the QLQ‐C30 scale. The median summary score of all patients with advanced cancer was 89.74, based on which the patients were classified into low or high QoL groups (Table S2). Logistic analysis showed that the WLAIGS was also a negative factor that independently affected patients' QoL. The risk of leading poorer QoL increased from 1.22 times in grade 2 to 1.88 times in grade 3 and 2.64 times in grade 4.

**TABLE 4:** Logistic regression analysis of WLAIGS associated with secondary outcomes

WLAIGS	Crude model		Adjusted model^a^		Adjusted model^b^	
	OR (95% CI)	*P* value	OR (95% CI)	*P* value	OR (95% CI)	*P* value
Short‐term survival						
Grade 1	Reference		Reference		Reference	
Grade 2	2.33 (1.85–2.94)	<0.001*	1.99 (1.58–2.53)	<0.001*	1.74 (1.37–2.22)	<0.001*
Grade 3	4.13 (3.32–5.16)	<0.001*	3.36 (2.69–4.22)	<0.001*	2.79 (2.22–3.52)	<0.001*
Grade 4	7.93 (6.36–9.93)	<0.001*	5.83 (4.65–7.33)	<0.001*	3.87 (3.02–4.98)	<0.001*
*P* for trend		<0.001*		<0.001*		<0.001*
Malnutrition (PGSGA)						
Grade 1	Reference		Reference		Reference	
Grade 2	2.47 (2.24–2.73)	<0.001*	2.16 (1.95–2.40)	<0.001*	1.49 (1.33–1.68)	<0.001*
Grade 3	3.56 (3.17–4.00)	<0.001*	3.24 (2.87–3.66)	<0.001*	2.34 (2.06–2.67)	<0.001*
Grade 4	23.28 (17.74–31.26)	<0.001*	20.24 (15.35–27.28)	<0.001*	7.16 (5.35–9.79)	<0.001*
*P* for trend		<0.001*		<0.001*		<0.001*
Quality of life						
Grade 1	Reference		Reference		Reference	
Grade 2	1.62 (1.48–1.78)	<0.001*	1.53 (1.39–1.69)	<0.001*	1.22 (1.09–1.37)	0.001*
Grade 3	2.61 (2.36–2.89)	<0.001*	2.39 (2.15–2.65)	<0.001*	1.88 (1.65–2.13)	<0.001*
Grade 4	5.70 (4.96–6.56)	<0.001*	5.07 (4.40–5.85)	<0.001*	2.64 (2.21–3.15)	<0.001*
*P* for trend		<0.001*		<0.001*		<0.001*

The Wald test was used to test the significance of each variable included in the logistic regression.

CI, confidence interval; OR, odds ratio; WLAIGS, weight loss and inflammation grading system.

^a^ Adjusted for age, sex, TNM stage, and tumour type.

^b^ Adjusted for age, sex, smoking, drinking, diabetes, hypertension, tumour type, TNM stage, treatments, NRS 2002, ECOG, BMI, CC, and HGS.

### Two internal validation cohort analyses

According to a 7:3 ratio, all advanced cancer patients were divided into two internal validation cohorts, with 7999 and 3424 participants in cohorts A and B, respectively. Patient demographic information, tumour‐related data, and laboratory tests between the two groups are shown in Table S3. The proportions of patients with grade 1 to grade 4 WLAIGS were 43.1%, 25.3%, 19.6%, and 12.0%, respectively, in cohort A and 43.8%, 24.3%, 20.5% and 11.3%, respectively, in cohort B. Univariate and multivariate Cox regression analyses of OS were performed in the two groups. After adjusting for confounders, the WLAIGS was confirmed to be an independent unfavourable factor for survival in both internal validation cohorts (Table S4). Compared with grade 1, the HRs of grade 2, 3 and 4 were 1.16 (95% CI, 1.06–1.27; *P* = 0.001), 1.49 (95% CI, 1.36–1.63; *P* < 0.001) and 1.72 (95% CI, 1.54–1.91; *P* < 0.001), respectively, in cohort A and 1.30 (95% CI, 1.13–1.49; *P* < 0.001), 1.51 (95% CI, 1.31–1.74; *P* < 0.001) and 1.84 (95% CI, 1.55–2.18; *P* < 0.001), respectively, in cohort B.

## Discussion

Weight loss has been recognized as a negative prognostic predictor in patients with cancer, independent of other factors, including age, sex, tumour site, and TNM stage.^1^ Martin et al. developed a robust BMI‐adjusted weight loss grading system for survival prediction in patients with cancer, and confirmed the severity of weight loss should be evaluated based on the rate of weight loss and the level of depletion of body reserves.^23^ However, in addition to initial body reserves, systemic inflammation response is another essential factor that should be considered during involuntary weight loss.^27^ Cancer‐related chronic inflammation promotes tissue catabolism, increases energy expenditure, and inhibits nutrient utilization, ultimately leading to the involuntary loss of body weight.^10^ In another international multi‐cohort study conducted by Martin et al., they further confirmed that elevated level of inflammation was independently associated with involuntary weight loss, with the risk rising to more than 2 times in patients with C‐reactive protein (CRP) above 10 mg/L adjusted for food intake.^28^ Moreover, they reported that high CRP level and weight loss grade were independent factors negatively affecting the survival of patients with cancer. Kaegi–Braun's findings indicated that inflammation showed a stronger prognostic value compared with other criteria of modified Global Leadership Initiative Malnutrition (mGLIM).^29^ Therefore, we speculated that the prognosis of cancer patients with approximate weight loss degree, but different inflammatory level may differ. In contrast to weight loss caused by reduced food intake alone, inflammation‐related weight loss may be associated with worse prognosis and may not be completely reversed by nutritional supplementation alone. In the present study, for the first time, we developed a novel cancer‐related prognostic grading system incorporating two factors: per cent weight loss and NLR. We first divided the patients into four groups according to their per cent weight loss, with cut‐off values of 2.5, 6.0, and 11.0. Each group was then divided into four subgroups according to the inflammation status. A 4 × 4 matrix representing four different percentages of weight loss categories within each of the four NLR categories (16 possible combinations of weight loss and NLR) was constructed. We calculated the HRs associated with OS for each group with reference to Group 1 (weight loss <2.5 and NLR ≤ 3). Groups with approximate HRs were classified into one grade, with the survival rate decreasing from grades 1 to 4. Comprehensive survival analyses were performed to investigate the prognostic value of the WLAIGS in patients with advanced cancer. The association between the WLAIGS and secondary outcomes, including short‐term survival, malnutrition, and QoL, was also explored.

Considering cure in most cases of advanced cancer remains difficult, an effective prognostic grading system may be beneficial for prognostic stratification and timely therapeutic interventions in clinical practice. In comparison to cancer patients with early stage, advanced patients exhibit a higher risk of weight loss.^30^ Moreover, the presence of an advanced cancer frequently correlates with systemic inflammatory burden, which, in turn, promotes catabolism and ultimately leads to involuntary weight loss.^10^ In addition, cancer patients with advanced stage were required to receive longer systemic anti‐cancer treatments, which may also lead to body weight loss and increased inflammatory level.^31^ In the present study, we found that patients with cancer in the advanced stages lost more body weight and had a higher inflammatory status than those in the early stages. This is consistent with previous data, which reported that weight loss was among the first symptoms in more than 50% advanced cancer patients and rose to more than 70% during the further course of the disease.^10^ Similarly, there also exists evidence that the NLR level increases with tumour stage.^14^ Another finding of our study was that the HRs of survival was in a general upward trend as the level of NLR increases in patients with approximate weight loss. In addition, the grades of WLAIGS would not surpass grade 2 if patients experience NLR ≤ 3. This indicates that weight loss without inflammation is associated with a relatively good prognosis. The results of the subgroup analyses confirmed that a high inflammatory status independently predicted worse survival of patients in the same weight loss group. Laviano et al. also reported that disease‐resulting weight loss, along with alterations in body metabolism and composition, had a more profound effect on patient outcomes than weight loss caused by simple starvation.^32^ Moreover, changes in body weight may appear behind internal metabolic alterations. Our study showed that a high NLR and a slight loss of body weight were also associated with worse survival. In short, the WLAIGS can not only distinguish the prognosis of patients with weight loss with/without inflammation but also predict survival before the presence of obvious weight loss. In clinical practice, appropriate anti‐inflammatory therapy may be recommended for patients with a high systemic inflammatory response.

This novel cancer‐related grading system consists of two powerful prognostic predictors: per cent weight loss and the NLR, which aligns with the GLIM criteria. As single components of the criteria, both long‐term weight loss and inflammation have been reported to be associated with adverse clinical outcomes, mortality or functional decline.^29^ We further performed survival analysis to investigate the prognostic value of the WLAIGS in patients with advanced cancer. The Kaplan–Meier curve showed that WLAIGS had a powerful step‐down prognostic stratification, with the survival probability decreasing from grade 1 to grade 4. Multivariate Cox regression analysis showed that WLAIGS was confirmed as an independent risk factor associated with OS after adjusting for confounders. With reference to grade 1, the prognostic risk from grade 2 to grade 4 showed a gradual upward trend. The results were the same in the sensitivity analysis, excluding patients who died within 6 months to avoid a cause‐reverse effect. In the tumour‐specific analysis, we found that the prognostic discrimination performance of the WLAIGS with four grades was significant in patients with advanced lung cancer. A similar trend was observed for other tumour types; however, it was not statistically significant for all grades. This may be partly because patients with lung cancer are more likely to experience inflammation‐related weight loss, whereas weight loss in patients with gastrointestinal cancer is more frequently affected by reduced food intake. Prospective large‐scale clinical studies on a single tumour type are needed to investigate the prognostic effect of WLAIGS. In addition, we further confirmed the prognostic performance of WLAIGS for patients in stage III and IV using subgroup analysis. We also divided all participants into two internal validation cohorts at a ratio of 7:3 to validate the prognostic value of the WLAIGS in patients with advanced cancer. The results of the present study are consistent with those of Fearon et al. They reported that a three‐factor profile incorporating weight loss, reduced food intake, and systemic inflammation identified both adverse function and poor prognosis in patients with cancer cachexia.^33^

To comprehensively investigate the effect of WLAIGS on prognosis, we also set various secondary outcomes, including short‐term survival, malnutrition, and QoL. A secondary analysis of a randomized clinical trial reported that mGLIM criteria served as a strong predictor for short‐term adverse clinical outcomes.^29^ In line with previous study, we found that WLAIGS was not only an independent unfavourable factor associated with OS but also negatively affected short‐term survival. The risk of dying at 90 days increased by more than 1, 2, and 3 times in grades 2 to 4 compared with grade 1. Rocha et al. found that the combination of modified Glasgow Prognostic Score and ECOG‐PS was beneficial in predicting survival at 3 months. It may be recommended for short‐term survival prediction in cases where weight loss is not present.^34^ We assessed patients' nutritional status using PG‐SGA, those who with a score equal to or more than four were diagnosed with malnutrition. After adjusting for confounders, the WLAIGS was confirmed as an independent risk factor for malnutrition, with ORs increasing from grade 2 to grade 4. The QoL of patients with cancer has attracted great attention owing to the extension of life.^35^ The QLQ‐C30 is a useful tool in clinical practice for assessing the QoL. In our study, we found that patients in grade 4 had significantly lower daily functions, including physical, role, emotional, cognitive, and social functions, as well as more frequent clinical symptoms. We divided all patients into low‐ and high‐QoL groups, with a cut‐off value of the median summary score for all participants. Logistic regression analysis showed that the risk of poor QoL increased with the WLAIGS grade in patients with advanced cancer.

This study has some limitations. First, all participants were from medical centers in China, and the prognostic performance of the WLAIGS in populations beyond China needs to be validated. Second, as an observational study, unmeasured or unknown confounding variables may have affected the outcomes. Third, although we used two internal validation cohorts to confirm our results, external validation analysis and randomized controlled clinical trials are required.

In conclusion, WLAIGS is a convenient and powerful grading system for predicting the prognosis of patients with advanced cancer. In patients with similar weight loss, a high inflammatory burden was associated with shorter survival.

## Conflict of interest

None declared.

## Funding

This work was supported by the National Multidisciplinary Cooperative Diagnosis and Treatment Capacity Project for Major Diseases: Comprehensive Treatment and Management of Critically Ill Elderly Inpatients (No. 2019.YLFW).

## Supporting information


**Table S1.** The weight loss and inflammation grading system (WLAIGS).
**Table S2.** Evaluation quality of life using QLQ‐C30 in different grades of WLAIGS.
**Table S3.** Baseline characteristics in two internal validation cohorts.
**Table S4.** Univariate and multivariate Cox regression in two internal validation cohorts.
**Figure S1.** Flowchart of the present study.
**Figure S2.** The prognostic value of WLAIGS in tumour‐specific analysis.
**Figure S3.** Sensitive analysis excluding patients who died within 6 months.
**Figure S4.** Kaplan–Meier curves between patients with/without inflammation.
