# Supplementary material for: A promising prognostic grading system incorporating weight loss and inflammation in patients with advanced cancer
Source: J Cachexia Sarcopenia Muscle. 2023 Nov 20;14(6):2969–80. doi: 10.1002/jcsm.13376 (PMC10751406; doi:10.1002/jcsm.13376)

**TABLE S1** The weight loss and inflammation grading system (WLAIGS)

| Weight loss (%) | Neutrophil to lymphocyte ratio (NLR) | | | |
| --- | --- | --- | --- | --- |
|  | ≤3 | (3,5] | (5,10] | >10 |
| <2.5 | 1 | 2 | 3 | 3 |
| [2.5,6) | 1 | 3 | 3 | 4 |
| [6,11) | 2 | 3 | 4 | 4 |
| ≥11 | 2 | 4 | 4 | 4 |

**TABLE S2** Evaluation quality of life using QLQ-C30 in different grades of WLAIGS

| **Variables** | **All patients**  **(n=11423)** | **Grade 1**  **(n=4951)** | **Grade 2**  **(n=2854)** | **Grade 3**  **(n=2270)** | **Grade 4**  **(n=1348)** | **P value** |
| --- | --- | --- | --- | --- | --- | --- |
| Physical function | 86.67(73.33-100.00) | 93.33(86.67-100.00) | 86.67(80.00-100.00) | 86.67(66.67-100.00) | 73.33(46.67-86.67) | <0.001* |
| Role function | 83.33(66.67-100.00) | 100.00(66.67-100.00) | 83.33(66.67-100.00) | 66.67(66.67-100.00) | 66.67(33.33-100.00) | <0.001* |
| Emotional function | 100.00(83.33-100.00) | 100.00(83.33-100.00) | 100.00(83.33-100.00) | 91.67(75.00-100.00) | 83.33(66.67-100.00) | <0.001* |
| Cognitive function | 100.00(83.33-100.00) | 100.00(83.33-100.00) | 100.00(83.33-100.00) | 100.00(83.33-100.00) | 83.33(66.67-100.00) | <0.001* |
| Social function | 66.67(66.67-100.00) | 66.67(66.67-100.00) | 66.67(66.67-100.00) | 66.67(66.67-100.00) | 66.67(33.33-66.67) | <0.001* |
| Global quality of life | 66.67(50.00-83.33) | 66.67(50.00-83.33) | 66.67(50.00-75.00) | 66.67(50.00-66.67) | 50.00(33.33-66.67) | <0.001* |
| Fatigue | 11.11(0.00-33.33) | 0.00(0.00-22.22) | 11.11(0.00-33.33) | 22.22(0.00-33.33) | 33.33(11.11-44.44) | <0.001* |
| Nausea and vomiting | 0.00(0.00-0.00) | 0.00(0.00-0.00) | 0.00(0.00-0.00) | 0.00(0.00-0.00) | 0.00(0.00-16.67) | <0.001* |
| Pain | 0.00(0.00-16.67) | 0.00(0.00-16.67) | 0.00(0.00-16.67) | 0.00(0.00-33.33) | 16.67(0.00-33.33) | <0.001* |
| Dyspnea | 0.00(0.00-0.00) | 0.00(0.00-0.00) | 0.00(0.00-0.00) | 0.00(0.00-33.33) | 0.00(0.00-33.33) | <0.001* |
| Insomnia | 0.00(0.00-33.33) | 0.00(0.00-33.33) | 0.00(0.00-33.33) | 0.00(0.00-33.33) | 33.33(0.00-33.33) | <0.001* |
| Loss of appetite | 0.00(0.00-33.33) | 0.00(0.00-0.00) | 0.00(0.00-33.33) | 0.00(0.00-33.33) | 33.33(0.00-33.33) | <0.001* |
| Constipation | 0.00(0.00-0.00) | 0.00(0.00-0.00) | 0.00(0.00-0.00) | 0.00(0.00-0.00) | 0.00(0.00-33.33) | <0.001* |
| Diarrhea | 0.00(0.00-0.00) | 0.00(0.00-0.00) | 0.00(0.00-0.00) | 0.00(0.00-0.00) | 0.00(0.00-0.00) | <0.001* |
| Financial impact | 33.33(0.00-33.33) | 33.33(0.00-33.33) | 33.33(0.00-33.33) | 33.33(0.00-66.67) | 33.33(33.33-66.67) | <0.001* |
| Summary score | 89.74(80.64-95.68) | 92.74(85.56-97.44) | 89.91(81.28-95.64) | 87.01(77.36-93.85) | 79.51(66.31-88.72) | <0.001* |

Abbreviation: QLQ-C30, 30-item European Organization for Research and Treatment of Cancer Quality of Life Questionnaire, version 3.0; WLAIGS, weight loss and inflammation grading system.

**TABLE S3** Baseline characteristics in two internal validation cohorts

| **Variables** | **Validation cohort A**  **(n=7999)** | **Validation cohort B**  **(n=3424)** | **P value** |
| --- | --- | --- | --- |
| Age, years | 59.00(50.00-66.00) | 59.00(50.00-66.00) | 0.812 |
| Gender (Male/Female) | 4817/3182(60.2%/39.8%) | 2060/1364(60.2%/39.8%) | 0.972 |
| Smoking (Yes/No) | 3598/4401(45.0%/55.0%) | 1539/1885(44.9%/55.1%) | 0.990 |
| Drinking (Yes/No) | 1634/6365(20.4%/79.6%) | 712/2712(20.8%/79.2%) | 0.675 |
| Diabetes (Yes/No) | 703/7296(8.8%/91.2%) | 272/3152(7.9%/92.1%) | 0.149 |
| Hypertension (Yes/No) | 1385/6614(17.3%/82.7%) | 625/2799(18.3%/81.7%) | 0.238 |
| Type of cancer |  |  | 0.721 |
| Lung | 2083(26.0%) | 868(25.4%) |  |
| Upper gastrointestinal | 1569(19.6%) | 667(19.5%) |  |
| Hepatobiliary and pancreatic | 410(5.1%) | 161(4.7%) |  |
| Colorectal | 1501(18.8%) | 658(19.2%) |  |
| Others | 2436(30.5%) | 1070(31.2%) |  |
| TNM stage |  |  | 1.000 |
| III | 3200(40.0%) | 1370(40.0%) |  |
| IV | 4799(60.0%) | 2054(60.0%) |  |
| Surgery (Yes/No) | 3059/4940(38.2%/61.8%) | 1283/2141(37.5%/62.5%) | 0.449 |
| Radiotherapy (Yes/No) | 1179/6820(14.7%/85.3%) | 506/2918(14.8%/85.2%) | 0.980 |
| Chemotherapy (Yes/No) | 3967/4032(49.6%/50.4%) | 1665/1759(48.6%/51.4%) | 0.354 |
| NRS2002(＜3/≥3) | 5185/2814(64.8%/35.2%) | 2209/1215(64.5%/35.5%) | 0.771 |
| ECOG grade(≤1/＞1) | 3478/4521(43.5%/56.5%) | 1580/1844(46.1%/53.9%) | 0.009* |
| PGSGA(＜4/≥4) | 2580/5419(32.3%/67.7%) | 1118/2306(32.7%/67.3%) | 0.693 |
| Body Mass Index, kg/m² | 22.04(19.82-24.26) | 21.97(19.84-24.38) | 0.945 |
| Calf circumference, cm | 32.50(30.20-35.00) | 33.00(30.40-35.00) | 0.097 |
| Hand grip strength, kg | 24.00(17.70-31.00) | 24.00(18.00-31.20) | 0.190 |
| White blood cell, X${10}^{9}$/L | 6.10(4.70-7.97) | 6.10(4.70-7.89) | 0.297 |
| Neutrophil, X${10}^{9}$/L | 3.86(2.71-5.54) | 3.83(2.71-5.42) | 0.354 |
| Lymphocyte, X${10}^{9}$/L | 1.40(1.00-1.84) | 1.40(0.98-1.85) | 0.424 |
| NLR | 2.70(1.81-4.42) | 2.68(1.79-4.48) | 0.492 |
| WLAIGS |  |  | 0.390 |
| Grade 1 | 3450(43.1%) | 1501(43.8%) |  |
| Grade 2 | 2021(25.3%) | 833(24.3%) |  |
| Grade 3 | 1568(19.6%) | 702(20.5%) |  |
| Grade 4 | 960(12.0%) | 388(11.3%) |  |

Abbreviations: WLAIGS, weight loss and inflammation grading system; TNM, tumor/node/metastasis; NRS 2002, Nutrition Risk Screen 2002; ECOG, Eastern Cooperative Oncology Group, PG-SGA, Patient-Generated Subjective Global Assessment; NLR, neutrophils to lymphocyte ratio.

**TABLE S4** Univariate and multivariate Cox regression in two internal validation cohorts

| **Variables** | **Validation A** | | | | |  | **Validation B** | | | | |
| --- | --- | --- | --- | --- | --- | --- | --- | --- | --- | --- | --- |
|  | **Univariate** | |  | **Multivariate** | |  | **Univariate** | |  | **Multivariate** | |
|  | **HR(95%CI)** | **P value** |  | **HR(95%CI)** | **P value** |  | **HR(95%CI)** | **P value** |  | **HR(95%CI)** | **P value** |
| Age, years |  |  |  |  |  |  |  |  |  |  |  |
| ≤65 | Reference |  |  |  |  |  | Reference |  |  |  |  |
| ＞65 | 1.50(1.40-1.61) | ＜0.001* |  | 1.18(1.09-1.27) | ＜0.001* |  | 1.59(1.43-1.77) | ＜0.001* |  | 1.34(1.20-1.50) | ＜0.001* |
| Gender |  |  |  |  |  |  |  |  |  |  |  |
| Female | Reference |  |  |  |  |  | Reference |  |  |  |  |
| Male | 1.40(1.31-1.50) | ＜0.001* |  | 1.10(1.00-1.20) | 0.047* |  | 1.40(1.26-1.55) | ＜0.001* |  | 1.16(1.01-1.33) | 0.042* |
| Smoking |  |  |  |  |  |  |  |  |  |  |  |
| No | Reference |  |  |  |  |  | Reference |  |  |  |  |
| Yes | 1.41(1.32-1.50) | ＜0.001* |  | 1.08(0.99-1.18) | 0.071 |  | 1.41(1.28-1.57) | ＜0.001* |  | 1.12(0.98-1.29) | 0.093 |
| Drinking |  |  |  |  |  |  |  |  |  |  |  |
| No | Reference |  |  |  |  |  | Reference |  |  |  |  |
| Yes | 1.28(1.18-1.38) | ＜0.001* |  | 1.10(1.01-1.20) | 0.034* |  | 1.25(1.10-1.40) | ＜0.001* |  | 0.99(0.87-1.14) | 0.907 |
| Diabetes |  |  |  |  |  |  |  |  |  |  |  |
| No | Reference |  |  |  |  |  | Reference |  |  |  |  |
| Yes | 1.18(1.06-1.32) | 0.003* |  | 1.07(0.95-1.19) | 0.274 |  | 1.32(1.11-1.57) | 0.016* |  | 1.14(0.95-1.37) | 0.146 |
| Hypertension |  |  |  |  |  |  |  |  |  |  |  |
| No | Reference |  |  |  |  |  | Reference |  |  |  |  |
| Yes | 1.09(1.00-1.18) | 0.061 |  |  |  |  | 1.14(1.00-1.29) | 0.048* |  | 0.95(0.83-1.08) | 0.426 |
| Type of cancer |  |  |  |  |  |  |  |  |  |  |  |
| Lung | Reference |  |  |  |  |  | Reference |  |  |  |  |
| Upper Gastrointestinal | 0.90(0.83-0.98) | 0.019* |  | 1.01(0.92-1.11) | 0.848 |  | 0.82(0.72-0.94) | 0.003* |  | 0.91(0.79-1.06) | 0.216 |
| Hepatobiliary and pancreatic | 1.19(1.03-1.36) | 0.015* |  | 1.26(1.09-1.45) | 0.001* |  | 1.21(0.98-1.49) | 0.077 |  | 1.13(0.90-1.41) | 0.281 |
| Colorectal | 0.50(0.46-0.56) | ＜0.001* |  | 0.65(0.58-0.72) | ＜0.001* |  | 0.48(0.41-0.56) | ＜0.001* |  | 0.59(0.50-0.70) | ＜0.001* |
| Others | 0.32(0.29-0.35) | ＜0.001* |  | 0.42(0.38-0.46) | ＜0.001* |  | 0.31(0.27-0.36) | ＜0.001* |  | 0.42(0.36-0.49) | ＜0.001* |
| TNM stage |  |  |  |  |  |  |  |  |  |  |  |
| III | Reference |  |  |  |  |  | Reference |  |  |  |  |
| IV | 2.23(2.07-2.39) | ＜0.001* |  | 1.87(1.74-2.02) | ＜0.001* |  | 2.65(2.36-2.98) | ＜0.001* |  | 2.13(1.89-2.40) | ＜0.001* |
| Surgery |  |  |  |  |  |  |  |  |  |  |  |
| No | Reference |  |  |  |  |  | Reference |  |  |  |  |
| Yes | 0.81(0.76-0.87) | ＜0.001* |  | 0.86(0.79-0.92) | ＜0.001* |  | 0.83(0.74-0.92) | ＜0.001* |  | 0.93(0.83-1.05) | 0.245 |
| Radiotherapy |  |  |  |  |  |  |  |  |  |  |  |
| No | Reference |  |  |  |  |  | Reference |  |  |  |  |
| Yes | 1.09(1.00-1.19) | 0.057 |  |  |  |  | 1.14(0.99-1.30) | 0.063 |  |  |  |
| Chemotherapy |  |  |  |  |  |  |  |  |  |  |  |
| No | Reference |  |  |  |  |  | Reference |  |  |  |  |
| Yes | 1.23(1.15-1.32) | ＜0.001* |  | 1.28(1.19-1.37) | ＜0.001* |  | 1.31(1.18-1.45) | ＜0.001* |  | 1.40(1.26-1.56) | ＜0.001* |
| NRS2002 |  |  |  |  |  |  |  |  |  |  |  |
| ＜3 | Reference |  |  |  |  |  | Reference |  |  |  |  |
| ≥3 | 1.66(1.56-1.78) | ＜0.001* |  | 1.15(1.06-1.25) | ＜0.001* |  | 1.61(1.46-1.79) | ＜0.001* |  | 1.04(0.92-1.18) | 0.556 |
| ECOG grade |  |  |  |  |  |  |  |  |  |  |  |
| ≤1 | Reference |  |  |  |  |  | Reference |  |  |  |  |
| ＞1 | 1.88(1.75-2.02) | ＜0.001* |  | 1.51(1.41-1.63) | ＜0.001* |  | 1.81(1.62-2.01) | ＜0.001* |  | 1.39(1.25-1.56) | ＜0.001* |
| Body mass index, kg/m² |  |  |  |  |  |  |  |  |  |  |  |
| High(≥18.5) | Reference |  |  |  |  |  | Reference |  |  |  |  |
| Low(＜18.5) | 1.51(1.39-1.65) | ＜0.001* |  | 1.00(0.89-1.11) | 0.927 |  | 1.47(1.28-1.68) | ＜0.001* |  | 1.07(0.91-1.27) | 0.389 |
| Hand grip strength, kg |  |  |  |  |  |  |  |  |  |  |  |
| High(≥11.9) | Reference |  |  |  |  |  | Reference |  |  |  |  |
| Low(＜11.9) | 1.46(1.33-1.59) | ＜0.001* |  | 1.26(1.15-1.39) | ＜0.001* |  | 1.63(1.42-1.87) | ＜0.001* |  | 1.46(1.26-1.70) | ＜0.001* |
| Calf circumference, cm |  |  |  |  |  |  |  |  |  |  |  |
| High(≥33.2) | Reference |  |  |  |  |  | Reference |  |  |  |  |
| Low(＜33.2) | 1.48(1.37-1.60) | ＜0.001* |  | 1.13(1.04-1.24) | 0.006* |  | 1.44(1.27-1.62) | ＜0.001* |  | 0.99(0.86-1.14) | 0.881 |
| WLAIGS |  |  |  |  |  |  |  |  |  |  |  |
| Grade 1 | Reference |  |  |  |  |  | Reference |  |  |  |  |
| Grade 2 | 1.41(1.29-1.53) | ＜0.001* |  | 1.16(1.06-1.27) | 0.001* |  | 1.60(1.40-1.83) | ＜0.001* |  | 1.30(1.13-1.49) | ＜0.001* |
| Grade 3 | 1.91(1.75-2.09) | ＜0.001* |  | 1.49(1.36-1.63) | ＜0.001* |  | 1.97(1.72-2.26) | ＜0.001* |  | 1.51(1.31-1.74) | ＜0.001* |
| Grade 4 | 2.75(2.49-3.03) | ＜0.001* |  | 1.72(1.54-1.91) | ＜0.001* |  | 2.89(2.48-3.36) | ＜0.001* |  | 1.84(1.55-2.18) | ＜0.001* |

Those variables found significant at *P* < 0.05 in the univariate analyses were entered into the multivariate Cox regression analyses.

Abbreviation: HR, hazard ratio; CI, confidence interval; TNM, tumor/node/metastasis; NRS2002, Nutrition Risk Screen 2002; EOCG, Eastern Cooperative Oncology Group performance status; WLAIGS, weight loss and inflammation grading system.

**FIGURE S1** Flowchart of the present study


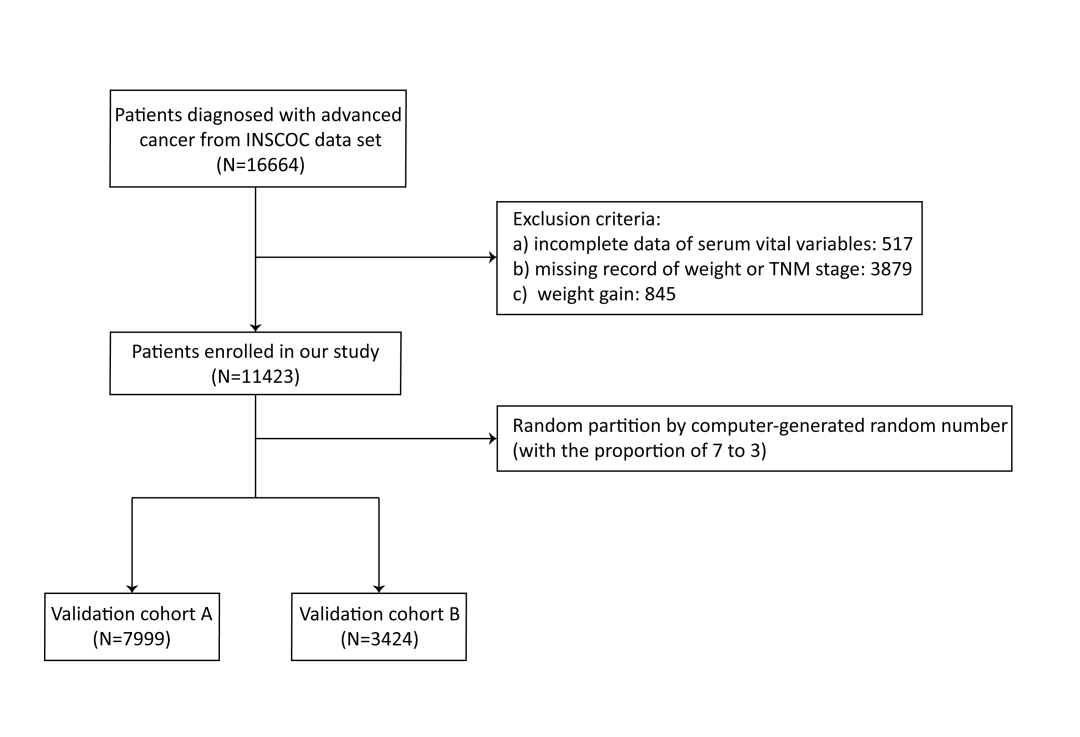


Abbreviation: INSCOC, Investigation on Nutrition Status and its Clinical Outcomes of Common Cancers project; TNM, tumor/node/metastasis.

**FIGURE S2** The prognostic value of WLAIGS in tumor-specific analysis


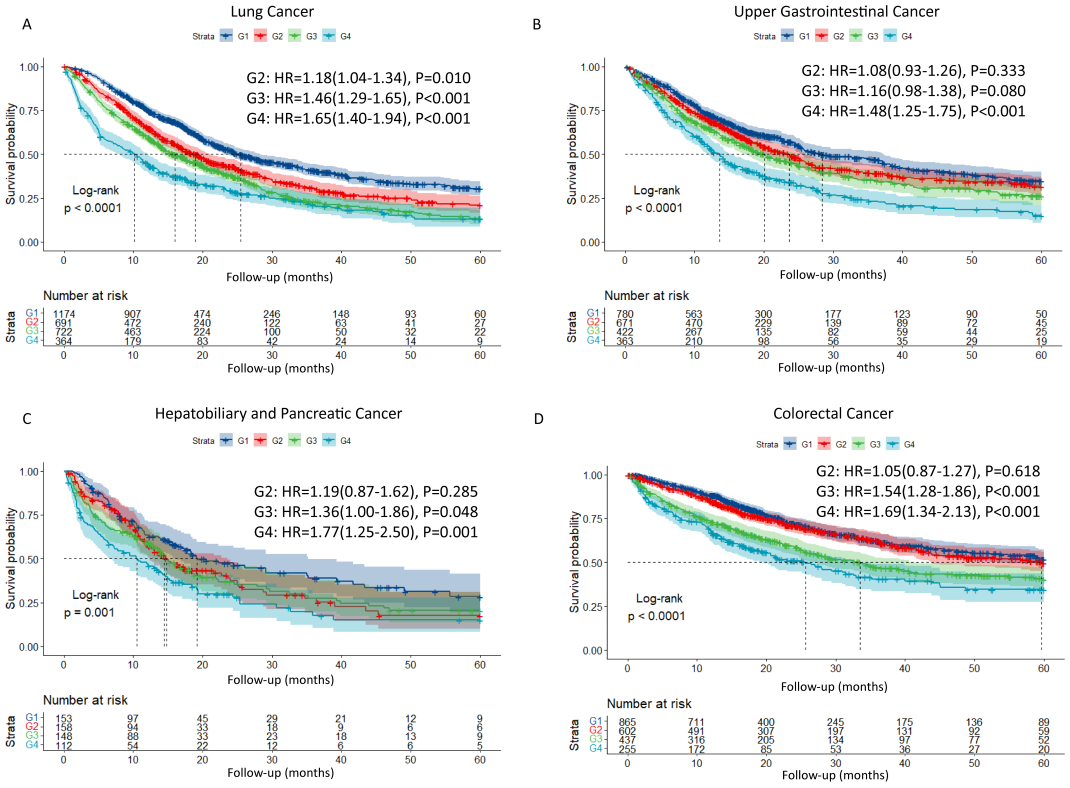


Abbreviation: HR: hazard ratio.

Adjust for variables found significant at P < 0.05 in the univariate analyses.

**FIGURE S3** Sensitive analysis excluding patients who died within 6 months


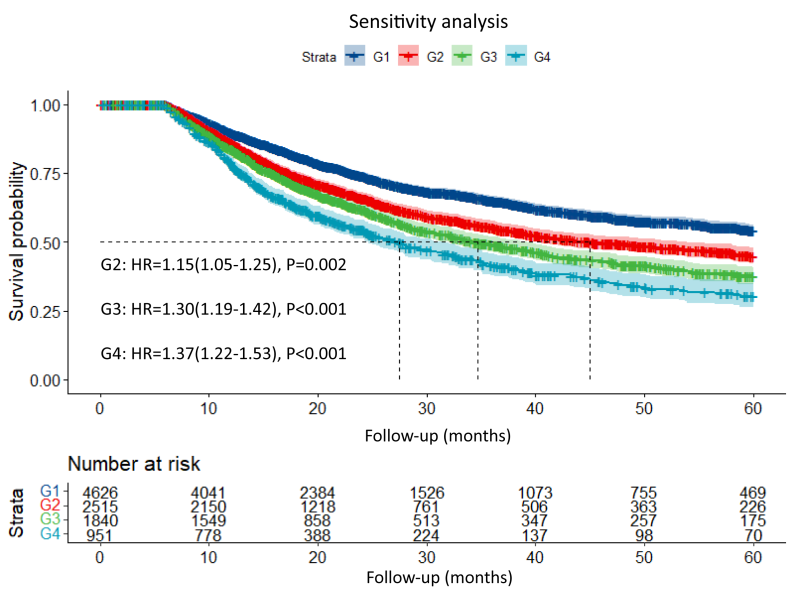


Abbreviation: HR: hazard ratio; OS, overall survival.

Adjusted for variables found significant at P < 0.05 in the univariate analyses, including age, sex, diabetes, smoking, drinking, tumor type, TNM stage, surgery, chemotherapy, NRS 2002, ECOG, BMI, HGS and CC.

**FIGURE S4** Kaplan-Meier curves between patients with/without inflammation


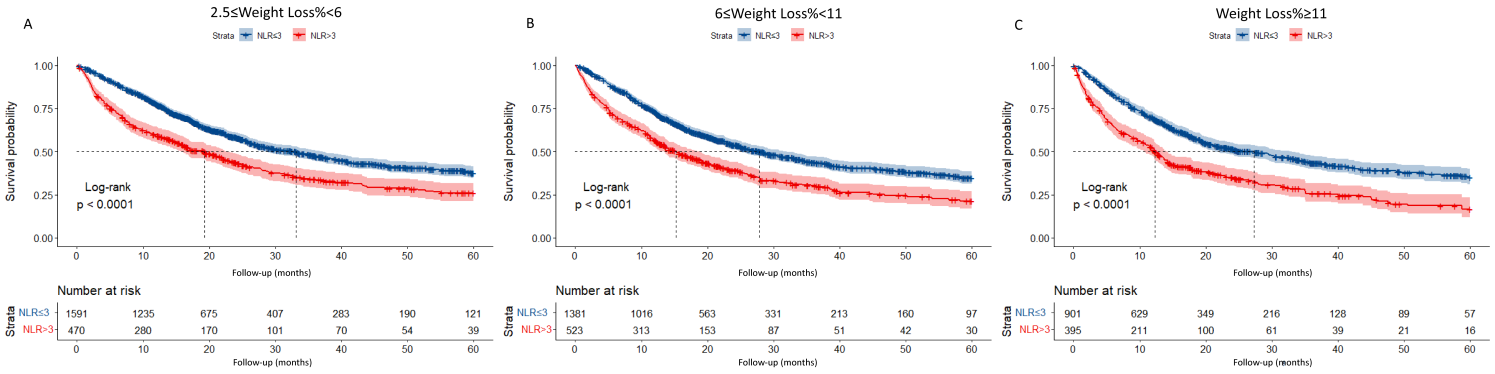

Supplement: Supplementary file 1 — Table S1. The weight loss and inflammation grading system (WLAIGS). Table S2. Evaluation quality of life using QLQ‐C30 in different grades of WLAIGS. Table S3. Baseline characteristics in two internal validation cohorts. Table S4. Univariate and multivariate Cox regression in two internal validation cohorts. Figure S1. Flowchart of the present study. Figure S2. The prognostic value of WLAIGS in tumour‐specific analysis. Figure S3. Sensitive analysis excluding patients who died within 6 months. Figure S4. Kaplan–Meier curves between patients with/without inflammation. [file JCSM-14-2969-s001.docx]
